# Healthcare professionals’ perspectives on oral health care in acute stroke patients: a qualitative study

**DOI:** 10.1038/s41405-024-00238-z

**Published:** 2024-06-19

**Authors:** Akua Boakyewaa Konadu, Emmanuel U. Iwuozo, Gifty Sunkwa-Mills, Yekeen A. Ayoola, Ewura A. Manu, Sandra A. Hewlett, Florence Dedey, Mohammed B. Abdulkadir, Olugbenga Ogedegbe

**Affiliations:** 1 Department of Restorative Dentistry, University of Ghana Dental School, Accra, Ghana; 2 Neurology Unit, Internal Medicine Department, Benue State University Teaching Hospital Makurdi, Makurdi, Benue State Nigeria; 3 Municipal Health Directorate, Awutu Senya East Municipal GHS, Awutu Senya East, Ghana; 4 Department of Medicine, Gombe State University, Gombe, Nigeria; 5 Department of Child Health, Korle Bu Teaching Hospital, Accra, Ghana; 6 Department of Surgery, University of Ghana Medical School, Accra, Ghana; 7 Department of Paediatrics, University of Ilorin, Ilorin, Nigeria; 8 Institute of Excellence in Health Equity, NYU Langone, New York, NY USA

**Keywords:** Special care dentistry, Oral conditions

## Abstract

**Background:**

Stroke is a major public health problem, with the disabilities of the patients increasing their risk of poor oral health. Currently, in Ghana and Nigeria, no guideline exists for oral health care in stroke patients, while most of our acute stroke care settings have no documented protocol.

**Aim:**

This study sought to understand the perspectives of healthcare professionals in Ghana and Nigeria about oral healthcare in acute stroke patients.

**Methods:**

A qualitative inductive approach was employed to explore healthcare professionals’ perspectives. After obtaining informed consent, in-depth interviews were conducted among doctors, nurses, and physiotherapists using semi-structured question guides. Participants’ responses were audiotaped for transcription and analysis. Interviews were conducted until data saturation was reached. Data were analyzed thematically to generate themes and sub-themes using an inductive approach.

**The results:**

Twenty-five (25) health care professionals (HCP) aged 25–60 years with a mean age of 36.2 ± 4.2 years were interviewed. There were 15 (60.0%) males and 10 (40.0%) females. HCP included nurse practitioners 14 (56.0%), medical doctors 7 (28.0%), and physiotherapists 4 (16.0%). The HCP demonstrated adequate knowledge of stroke and considered oral health important for esthetic and clinical reasons. They, however, reported being unable to give it the required attention due to inadequate collaboration between the various professional cadres, insufficient equipment, and a skewed focus on other clinical needs of the acute stroke patients.

**Conclusions:**

The findings indicate that HCPs perceived oral health care as very important among patients with acute stroke. However, they reported a lack of collaboration and integration of oral health care in routine stroke care as a major impediment.

## Introduction

Stroke is a major global public health problem that is occurring in an epidemic dimension in sub-Saharan Africa, including Ghana and Nigeria [1–4]. Globally, it is the second leading cause of death and the major leading cause of disability with about 5 million people said to have developed permanent disability [1, 5]. In Africa, the prevalence rate can reach 315 per 100,000 people, with a three-year mortality rate of up to 84% [2].

Incidentally, LMICs in Africa like Ghana and Nigeria, have a dearth of stroke specialists, scant resources for acute stroke care, and lack facilities for post-stroke rehabilitation [1, 4].

Disabilities in stroke survivors could impair swallowing, movement, communication, cognition, and sensation and may also cause depression. A stroke-related disability could negatively affect survivors’ oral health care due to tongue weakness, facial weakness, dysphagia, incoordination of upper limbs, and upper limb hemiparesis (paresis) or hemiplegia (paralysis) leading to inability to maintain adequate oral hygiene independently [6–8]. This may result in halitosis, drooling of saliva, and an increase in the bacterial load in the mouth with the attendant risk of aspiration pneumonia. Therefore, ensuring good oral health care is an enormous task among hospitalized stroke survivors. Oral health care is necessary to maintain the health of the mouth, teeth, tongue, and gums after stroke. This ideally should be part of the rehabilitative process in these patients as they have difficulty in carrying out oral health care themselves [9].

Previous researchers [8, 10] have documented that stroke patients have poor oral health status, which could in turn lead to a negative impact on their well-being and quality of life [11, 12]. However, despite the need for good oral healthcare in stroke survivors, little attention has been given to it by healthcare professionals, especially in the acute stroke care setting. Most times, emphasis has been placed on the recovery of functional impairments like loss of consciousness, dysphagia, and motor and speech or language deficits [13, 14]. In order to ensure good oral healthcare in stroke survivors,’ healthcare professionals will need to provide relevant education and care. Ferguson et al., in a qualitative study exploring nursing and allied health clinicians’ perspectives of oral health care after stroke in Sydney, Australia, noted that most participants reported inadequate resources and training to offer this service to hospitalized stroke patients [15]. Healthcare professionals are expected to play a key role in early identification, assessment, referral and rehabilitation of these patients. Currently, in Ghana and Nigeria, no guideline exists for oral health care in stroke patients, while most of our acute stroke care settings have no documented protocol. It, therefore, becomes imperative to understand the perspectives of healthcare professionals in these two countries about oral healthcare in acute stroke care settings. This will be necessary in developing an optimal protocol. Hence, this study was intended to qualitatively explore the perceptions and practices of healthcare professionals managing acute stroke patients in this area of care. We also expect the findings from this study to be relevant to policymakers and healthcare administrators aside from HCPs (doctors, nurses, physiotherapists)

## Methodology

### Study design

This qualitative study used an inductive approach to explore the perception and practices of health care professional oral health care of stroke patients managed in the acute stroke care setting [16].

### Study setting

The study was conducted at two tertiary hospitals in West Africa (Ghana and Nigeria) between April 2021 and August 2021. The hospitals are Korle Bu Teaching Hospital (KBTH) in Accra, Ghana, and Benue State University Teaching Hospital (BSUTH) in Makurdi, Benue State, Nigeria. The two hospitals have facilities for acute stroke care and rehabilitation.

## Participants and sampling procedure

The target population was members of the multi-disciplinary stroke team of health care professionals like doctors, nurses, and physiotherapists responsible for managing acute stroke patients in both hospitals’ emergency wards, stroke units, medical wards, and physiotherapy units. Participants were recruited through a purposive sampling method by the principal investigators, IEU in BSUTH and ABK in KBTH. The principal investigators made prior notification about the research at both sites using information leaflets. Willing healthcare professionals were allowed to participate in a one-off face-to-face in-depth interview using a semi-structured questionnaire. In BSUTH, 14 HCPs were recruited for the study, while in KBTH, 11 were recruited. However, about 8 nurses, 4 doctors, and 3 physiotherapists could not participate in the study from both sites because they were on their annual leave.

### Data collection

An overview of the study objectives, participation requirements, and investigators was provided to willing participants through the study information leaflet. The interested healthcare professionals were allowed to ask questions before completing the informed consent forms. The participants were not previously known to the interviewers at both sites. The interviews were conducted at both sites by experienced qualitative researchers—AJR in BSUTH and SMG in KBTH in the office of the principal investigators and the absence of a third party.

The interviewers used the adapted shortened version of Adam’s questionnaire to guide the interview [17]. In the course of the interview, strategies like repeating questions, prompting, and clarifying responses were employed. Each interview lasted between 7 and 10 min, and participants’ responses were audiotaped for subsequent transcribing and analysis. Field notes were also made. Interviews were conducted until data saturation was reached.

## Data analysis

Data collection and analysis were done concurrently until saturation was attained. Interviews were digitally recorded and professionally transcribed verbatim by one of the investigators (SMG). Transcripts were de-identified to maintain confidentiality, and grounded theory was used to analyze the data [16]. To ensure the credibility and trustworthiness of the result, triangulation was employed. Data were analyzed thematically based on the objectives of the study using an inductive approach [16]. The transcripts were uploaded to QSR N Vivo 12 to support coding and analysis. Similar codes were grouped into categories. The categories were then regrouped into sub-themes and themes. Similarities and differences between the two sites and categories of participants were explored, and we ensured the loop was closed. The themes and sub-themes generated were checked and harmonized by two other investigators (IEU and ABK) to ensure the coherence of ideas and credibility of the results.

## Results

### Socio-demographic characteristics of participants

**Fig. 1:**
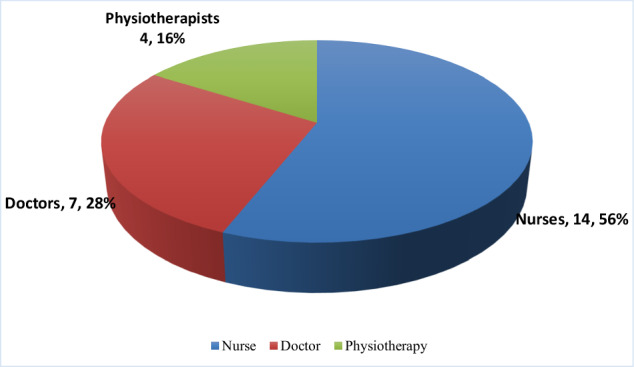
Distribution of healthcare professionals. Shows the healthcare professionals involved in stroke care who were interviewed.

We interviewed 25 healthcare professionals who are part of the multi-disciplinary team involved in the care of acute stroke patients. Their age ranged between 25 and 60 years, with a mean age of 36.2 ± 4.2 years. The majority of the participants were males; 15 (60.0%) and 10 (40.0%) were females. Also, the majority of the HCPs were registered nurse practitioners, 14 (56.0%), while 7 (28.0%) were medical doctors and 4 (16.0%) were physiotherapists, as shown in Fig. 1 below. Although the mean and median duration of practice of HCPs caring for acute stroke patients was 7.3 ± 2.3 and 4 years, respectively, this ranged from 4 months to 28 years.

### General knowledge of stoke and oral health in stroke

Healthcare professionals interviewed were experienced in the care of stroke patients and demonstrated adequate knowledge of stroke in general. They willingly described and discussed their expertise in stroke care and the associated or expected complications that stroke patients commonly encounter.

*As a clinician, I work in the stroke unit and with the neurology department. Stroke is the most common neurological condition we see, and we have a stroke unit. So when it comes to stroke, you see all kinds of stroke. In the stroke unit, about forty percent of them are haemorrhagic strokes, fifty percent are ischemic strokes, and we have a few subarachnoid haemorrhage, which is again part of the haemorrhagic stroke*. (Doctor, D1)

### Thematic analysis generated the following main themes

#### Clinical care is a priority for stroke patients

Healthcare professionals described the various types of stroke cases they attend to and the clinical care rendered to them. In the Korle-Bu Teaching Hospital, some HCPs (doctors, nurses, physiotherapists) attended to stroke patients in the Stroke Unit and some of the medical wards. HCPs (doctors, nurses, physiotherapists) described various aspects of care they considered critical for stroke patients. They also mentioned bed sores and deep vein thrombosis, which stroke patients are usually at risk of. While a few others were also worried about aspiration, stroke patients who are unable to swallow are prone to it.

*Most of them cannot swallow, so saliva and food can go the wrong way*. (Doctor, D2)

#### Oral health is essential to prevent health complications

All the HCPs interviewed mentioned oral health’s critical role in the journey of stroke patients toward recovery. They reported that they are concerned when patients have poor oral health, cannot chew, or cannot swallow, which is common in stroke patients.

Some statements by healthcare professionals are captured below to reflect this theme:

*Doctors usually check whether a patient can talk, chew & swallow, and if the patient cannot swallow, we fix nasogastric tube for feeding*. (Doctor, D3)

*Once an unconscious patient starts becoming aware of oral hygiene, we know there is an improvement*. (Nurse, N1)

*Aspiration is one of the things that we are worried about. So, when a patient comes and the assessment is done, we know that they cannot swallow saliva, so they are pooling. When they pool, it is a medium for bacteria to multiply. We have normal bacteria that live in the mouth, but if this goes down the throat to the lungs, they can have an infection. We check the swallowing, and if they cannot swallow, we check very often, but if they keep drooling, we know that the swallowing is impaired. We tend to position them upright or to their side so that they can drool freely. We also pass a nasogastric tube for feeding because sometimes it is not just the saliva that makes them aspirate*. (Doctor D3)

#### Oral health care is important for good oral health-related quality of life

Healthcare professionals mentioned the importance of oral health care in ensuring and preventing offensive odors among stroke patients. They also explained the importance of cleaning the mouth to prevent infection, dryness, and cracking. Prevention of bad odors or bad breath (halitosis) was emphasized as a motivation for paying attention to oral health.

*Generally, oral health has even been associated with depression because of bad breath (halitosis). Unfortunately, most people with halitosis do not even know they have it. Others do not say it to your face, but anytime you talk, they move away, depending on the type of stroke patient. If this is a stroke patient that has good cognition and he can tell that the odor coming out of the mouth is not good but has limb weakness involving his hand, then there is very little he can do to improve it. He has to rely on others to clean his mouth for him, and that can worsen the depression, which is usually high in people with stroke*. (Nurse N2)

*Some of them might get food pockets in their mouth. Some even come with Candida, especially in people with diabetes, when their oral care is inadequate. I must say it is not part of our core discharge plans, which we should be doing. So, they come back on review, and you realize that the person’s mouth is not being taken care of because,… Imagine brushing someone’s teeth, and you keep wondering how he will spit it out and all that. So those are the challenges we have*. (Doctor, D1)

#### Lack of collaboration and integration of oral health care in stroke care

Doctors and nurses agreed that health workers rarely collaborated to integrate oral health care into the patient’s care plan. In the stroke unit in KBTH, there appears to be some effort to implement a form of collaboration, but in practice, this was rarely achieved.

*Aside from the initial assessment, I can check when I ask the person to open the mouth, but the doctors hardly even look into the mouth. It is the nurses who do this when they clean the patient’s mouth*. (Doctor, D4)

Although HCPs (Doctors and Nurses) admitted to the importance of oral health in stroke, most of the doctors interviewed mentioned that they do not pay optimal attention to it.

*We do not do oral care as Doctors… Ours is to do a clinical evaluation while the nurses do oral and other care. We only guide… and it is not our role*. (Doctor, D5)

Although HCPs (Doctors and Nurses) feel inadequately equipped, some try to do their best, as they described.

*We are not dental surgeons, but we do our best. What we do as I said, the nurses use mouthwash, gauze, and a spatula and try as much as possible to get the debris and keep the place neat. That is what we do at the stroke unit*. (Doctor, D4)

#### Lack of standardization of tools for the provision of oral health care

The HCPs (Doctors and Nurses) mentioned lacking access to suitable oral healthcare assessment and protocol tools. The lack of this equipment and tools was mentioned as a critical barrier hindering oral health care.

*I recommend the use of mouthwash, which the patient has to buy*. (Doctor D6)

The HCPs (Doctors and Nurses) interviewed stated that the approaches toward assessing and caring for stroke patients’ oral needs varied between wards.

A doctor described that the initial assessment was sometimes not done well, setting a false preamble for further management.

*When it comes to aspiration, sometimes even the health care workers are the worst culprits. What usually happens at the emergency is that a doctor or nurse will give the patient a teaspoon of water (to check if they can swallow); if the patient can swallow, it is assumed that the patient is ok to move on to other food substances”… but swallowing isn’t just a teaspoon of water; so when the patient is given koko (porridge), and their swallowing muscles are fatigued, they can’t swallow*. (Doctor, D1)

*So you see someone you passed during the swallowing test, and the next moment, you find the patient down and febrile because we did not take the time to check well… that is something we need to be taught…. They have aspiration not because their family caused it but rather because we should have used more than just one and a half tablespoons of water to establish whether the patient is at risk of aspiration*. (Doctor, D1)

Furthermore, some HCPs (Doctors and Nurses) use flavored mouthwash for oral care, while others recommend an antibacterial-based mouthwash (such as chlorhexidine).

*Try and use chlorhexidine-based mouthwash and try to clean their tongue*. (Nurse N3)

Others further argued that the flavored mouthwash is ineffective and that one should use warm saline water, which is also soothing if there are ulcerations in the mouth.

*Instead of two times daily, you can do three times daily, and whenever you feed them, try to let them rinse their mouth; if they cannot, use gauze and a spatula so that you do not put your hand in there for them to bite you*. (Nurse, N4)

Nurses also mentioned that patients could use the toothbrush if they are able, but in those who are unconscious, we can use gauze, a spatula, and some toothpaste to clean the mouth, the teeth, and the tongue.

#### Promoting oral health in acute stroke patients

Healthcare professionals emphasized the need for multi-sectoral collaboration to promote oral health care among stroke patients. The importance of good nursing care was reiterated as nurses generally spend more time with the patient.

In KBTH, a doctor said:

*Nurses are trained to do this. It is their duty to provide day-to-day care for the patient every day*. (Doctor, D7)

A provisional arrangement was described where nurses received skill training and were supervised by doctors. He further explained that:

*Usually, when new personnel come in, they go through training on some of the modalities we have in the unit, so there is training on feeding and mood assessment and something like that. Once you have gone through the training, you do not really need supervision, but when we have multi-disciplinary rounds, and somebody is doing it, we are all able to assess, and if they are not doing it right, then we make an input* (Doctor, D7)

To strengthen the approach to promoting oral health care among stroke patients, healthcare personnel agreed that the multi-disciplinary approach to care should be institutionalized:

*During multi-disciplinary rounds, doctors will carry out their ward rounds, and nurses will give feedback on nursing care and any challenges they may encounter. Physiotherapy will also say if there are any challenges and what needs to be done better… If they haven’t started physiotherapy and why it hasn’t been started. The occupational therapist informs us about their assessment and whether they were able to visit the home. The speech therapist informs us of the patient’s abilities after they have started to evaluate the swallowing and speaking process. So, everyone makes an input, and we all make an input in the discharge planning and what should happen*. (Doctor, D2)

In a comprehensive discharge plan, preparation is made for the patient’s discharge and continued management at home. The process involves counseling the patient and preparing them for rehabilitation after the stroke. Helping patients and families to prepare food of the right consistency to prevent aspiration, how to feed and maintain hygiene, physical activities, and performing functions relevant to their functional recovery.

## Discussion

This qualitative study explored the perspectives of healthcare professionals (HCPs) caring for stroke patients regarding oral health care in Ghana and Nigeria. HCPs were knowledgeable about stroke in general and raised relevant concerns about oral health care for stroke patients. A previous study in Sydney, Australia, also revealed similar knowledge about stroke and its relationship with oral health among nurses and allied health stroke clinicians [15]. In that study, some participants were reported to have some misconceptions regarding oral care, such as that dentures make oral care easier and that those who choose to smoke do not care about their oral hygiene [15]. The HCPs considered clinical care of stroke patients as a major priority in the management of acute stroke patients and not oral health care. In general, it was agreed among HCPs in Ghana and Nigeria that oral care for stroke patients was usually neglected in the management of these patients. Therefore, there is a tendency to overlook oral care for patients with stroke, as corroborated by previous researchers [13, 14].

Also, HCPs in this study posited that good oral health hygiene is perceived as important among patients with stroke as it will lead to a reduction in the tendency to get infections from their mouth. This finding is consistent with previous documentation about the risk of aspiration pneumonia in stroke patients with poor oral health [18, 19]. The HCPs emphasized the importance of oral health care in ensuring and preventing offensive odors or bad breath (halitosis) among patients with stroke. Effective oral health care has been reported to prevent complications in stroke patients, promote oral comfort, and reduce halitosis [9].

We also discovered that a lack of cross-sector collaboration and integration of oral care in the care of acute stroke patients is a significant barrier to oral health care in our study. Oral health care was communicated differently and through separate communication channels by various healthcare professionals to patients and their caregivers. In this way, the issues regarding various aspects of oral hygiene were rarely addressed. In acute stroke care settings, oral health care was not included in patients’ clinical care plans, indicating an under-emphasis on it.

The lack of protocols for oral care during strokes was also highlighted as contributing to the low priority given to oral care in patients with stroke. The oral care practices in various wards and stroke care settings across the two countries differed considerably. Bangee and colleagues in the UK and Australia have also reported this variability in their study [14].

In Ghana, the guidelines at the stroke unit where this study was undertaken state that persons with stroke should be assisted in performing oral care three times daily to improve patient outcomes. There were no documented guidelines for oral health care for patients with stroke in Nigeria. Some previous researchers have also reported a lack of protocols for oral healthcare in the care of stroke patients [14, 15]. Although there isn't  much evidence for good oral healthcare in stroke patients, some previous researchers have recommended good clinical practice [11, 20–23]. It was suggested that guidelines on oral health care be generated and documented to help care for patients with stroke in Ghana and Nigeria.

It was clear that there was no standardization of oral healthcare tools. However, the study revealed that toothbrushes and antibacterial mouthwashes were used significantly in both countries when it came to tools and equipment available in both hospitals. Patients are responsible for providing basic products like manual toothbrushes and toothpaste in both hospitals. In Ghana, the stroke unit recommends the use of toothbrushes and toothpaste (with chlorhexidine-based mouthwash) as cleaning agents. Previous studies have shown that basic equipment such as toothbrushes and toothpaste are available in most hospitals, as well as various mouthwashes and rinses [14, 15]. Equally, some researchers have shown that certain rinses can maintain the oral mucosal membrane in good condition in critically ill patients [24]. There appears to be limited evidence to guide the choice of the best cleaning agents and tools to use in oral care in stroke [11, 25, 26]. It is imperative to provide the necessary orientation and tools for oral health care in the management of stroke patients.

Good clinical outcomes can be achieved by promoting oral health care in stroke patients to enhance good oral hygiene. Some guidelines have recommended that HCPs, especially nurses, should be educated and trained in oral health care [27, 28]. However, our study showed that the education and training of HCPs in oral health care were grossly inadequate. Previous studies have highlighted several barriers experienced, particularly by nurses, in delivering effective oral health care for patients with stroke. This included inadequate training, lack of knowledge of oral care resources, and excessive work overload [28–30]. Campbell et al. have shown that even an hour-long training session for HCPs by a dental health professional can improve staff knowledge and attitude about oral health care in stroke [12]. It has been suggested that relatives of acute stroke patients should also be educated and actively engaged in oral health care where healthcare professionals are unable to do so.

## Strengths and limitations

This study explored the perspectives of healthcare professionals from two hospitals (one in each country) in Ghana and Nigeria regarding oral healthcare for acute patients and provided insightful findings. The robust study design and methodology took into account the criteria for qualitative research, resulting in informative themes and subthemes. Some of the limitations could include the fact that some of the participants have little experience in caring for stroke patients, which could have an impact on their responses. Furthermore, the use of HCPs’ perceptions has been said not to be an accurate indicator of actual practice. Despite these limitations, the findings will be relevant in developing a guideline or protocol for oral health care in stroke patients in these facilities in Ghana and Nigeria.

## Recommendations

Oral health care could be improved by increasing training, performing individual assessments on admission, and using standardized assessment tools and protocols to guide high-quality care to improve patient outcomes.

## Conclusion

The findings from this study have highlighted considerable variability in the perceptions and practices of oral health care for acute stroke patients in Ghana and Nigeria. Oral care practices can be improved by training and orientation for staff, performing individual assessments for stroke patients on admission, and using standardized assessment protocols that inculcate oral care to ensure comprehensive, high-quality care. Oral health care interventions must be designed to be clinically feasible within stroke care settings. The delivery of oral health care interventions should be done in clinical contexts, which will facilitate translation into clinical practice. Further research could focus on the practicalities of incorporating oral care into existing clinical protocols and practices.

## Data Availability

The datasets generated and analyzed during the current study are not publicly available because we do not have consent from all patients to publish this data, and the data will need to be de-identified. However, they will be available from the corresponding author upon reasonable request.

## Appendix

**Table:**

**Interview Guide for Assessing health providers’ perspectives on Oral Health Care in Acute Stroke** The Shortened Version of the Adams’ Questionnaire Including the Correct Answers	
**Questions**	**Right Answers**
1. Can you tell me what you know about oral health and stroke?	Introducing the topic: Explore (The health of the teeth and gums in patients who have suffered a stroke)
2. How important is it to check the mouth of each stroke patient stroke on admission?	It is important because, if not done can predispose them to infections
3. What to assess to determine patients’ oral health status?	The patient’s chewing; swallowing and speaking functions should be assessed.
4. What are the necessary things to check out for in the patient’s oral cavity during the evaluation of oral hygiene status?	Presence of all-natural teeth Teeth health status Gingival health status Presence of oral mucosal lesions Presence, integrity and hygienic status of partial or
	complete denture Presence of oral pain and/or burning Presence of food debris/white patches
5. How many times a day should a patient’s oral health care be performed by health care personnel?	Twice daily
6. Which oral hygiene tools should be used to clean the patient’s teeth and oral mucosa or their dental prostheses?	Fluoride toothpaste Toothbrush Chlorhexidine mouthwash or gels Gauze soaked in the disinfectant solution for moisturizing the lips and washing dental prostheses.
**Notes:** Adapted with permission from Adams R. Qualified nurses lack adequate knowledge related to oral health, resulting in inadequate oral care of patients on medical wards. *J Adv Nurs*. 1996; 24(3):552–560. Copyright © John Wiley & Sons Ltd.18	
